# Reply to Response by Tariq to “Association Between Proton Pump Inhibitor Use and Short-term Postoperative Complications Following Abdominoplasty: A Multi-Institutional Cohort Study”

**DOI:** 10.1093/asjof/ojag201

**Published:** 2026-10-12

**Authors:** Yoram Wolf, Jonathan Zontag, Ron Skorochod, Nir Zontag

We appreciate the important commentary by Tariq et al on our recent article examining the association between perioperative use of proton pump inhibitor (PPI) medications and the risk for short-term postoperative complications among abdominoplasty candidates.^1^

In this study, we compared patients who were prescribed PPI in the year before surgery with control patients who were prescribed H2-receptor antagonists during the same timeframe, using TriNetX, a large-scale, worldwide database.^2^ The platform uses standardized, internationally validated coding systems for medications, procedures, diagnoses, and clinical outcomes, enabling assessment of diverse exposures and outcomes across large populations.^2^

Cohorts were balanced using 1:1 propensity score matching (PSM) with several covariates to enable appropriate comparison. Most variables matched well, except for a higher proportion of White individuals in the PPI cohort. We acknowledge that, as suggested by Tariq, reporting standardized mean differences (SMDs) for PSM may be preferable. In the TriNetX platform, PSM results are reported using both *P*-values and SMD. Both approaches consistently identified an imbalance in the proportion of White individuals among the unmatched participants, which was appropriately addressed in the limitations and the results sections as needed.

Although the database enables large-scale analyses and offers several well-established advantages, it also has inherent limitations. Specifically, the absence of detailed data on medication use, treatment regimens, adherence, medication switching, and other aspects of treatment exposure. As Tariq et al noted, this limitation prevents us from establishing causality. Therefore, these findings should be interpreted as hypothesis-generating, warranting further validation in future prospective studies with more granular clinical and medication-related data. In this study, we used a 1-year timeframe to identify patients prescribed PPI or H2-blocker medications. However, this timeframe may still encompass differences in treatment regimens, medication adherence, duration of use, and switching between medications.

## References

[1] Wolf Y, Zontag J, Skorochod R, Zontag N. Association between proton pump inhibitor use and short-term postoperative complications following abdominoplasty: a multi-institutional cohort study. Aesthetic Surg J Open Forum. 2026;8:ojag129. doi: 10.1093/asjof/ojag129 PMC13344097 42422341

[2] Palchuk MB, London JW, Perez-Rey D, et al A global federated real-world data and analytics platform for research. JAMIA Open. 2023;6:ooad035. doi: 10.1093/jamiaopen/ooad035 PMC10182857 37193038

